# Predictive Value of the C-Reactive Protein to Albumin Ratio in 30-Day Mortality after Hip Fracture in Elderly Population: A Retrospective Observational Cohort Study

**DOI:** 10.3390/jcm12134544

**Published:** 2023-07-07

**Authors:** Giorgio Cacciola, Fabio Mancino, Lukas A. Holzer, Federico De Meo, Ivan De Martino, Antongiulio Bruschetta, Salvatore Risitano, Luigi Sabatini, Pietro Cavaliere

**Affiliations:** 1 Orthopaedic Institute of Southern Italy “Franco Scalabrino”, 98165 Messina, Italy; 2 Department of Orthopaedics, The Orthopaedic Research Foundation of Western Australia (ORFWA), Fiona Stanley Fremantle Hospitals Group, Perth, WA 6150, Australia; 3 Università Cattolica del Sacro Cuore, Largo Francesco Vito, 1, 00168 Rome, Italy; 4 Adult Reconstruction and Joint Replacement Unit, Division of Sports Traumatology and Joint Replacement, Department of Ageing, Orthopaedic and Rheumatologic Sciences, Fondazione Policlinico Universitario Agostino Gemelli IRCCS, 00168 Rome, Italy; 5 A.O.U. Città della Salute e della Scienza, Centro Traumatologico Ortopedico (C.T.O.), Universitá di Torino, 10024 Turin, Italy

**Keywords:** femoral fractures, early mortality, C-reactive protein, albumin, arthroplasty, femoral nailing, intramedullary nail, total hip arthroplasty, elderly patients

## Abstract

Background: C-reactive protein (CRP) to Albumin ratio (CAR) has been used in multiple clinical settings to predict early mortality. However, there is a lack of evidence on the predictive role of CAR in 30-day mortality after a hip fracture. The purpose of this study was to establish a potential association between CAR and 30-day mortality and to assess if the CAR Receiving Operating Characteristics curve (ROC) can be a reliable predictor of early mortality. Methods: We retrospectively reviewed the charts of 676 patients (>65 years) treated for hip fracture between 2006 and 2018. All hip fractures were included. Treatment strategies included closed reduction and internal fixation, open reduction and internal fixation, hemiarthroplasty, or total joint arthroplasty. Statistical analysis included T-test, Pearson correlation for CAR and other markers, ROC curves and area under the curve, Youden Model, and Odds Ratio. Results: The 30-day mortality rate analysis showed that higher preoperative levels of CAR were associated with higher early mortality. When analyzing the area under the ROC curve (AUROC) for 30-day mortality, the reported value was 0.816. The point of the ROC curve corresponding to 14.72 was considered a cut-off with a specificity of 87% and a sensibility of 40.8%. When analyzing values higher than 14.72, the 30-day mortality rate was 17.9%, whilst, for values lower than 14.72, the 30-day mortality rate was 1.8%. Conclusions: Patients older than 65 years affected by a hip fracture with increased preoperative levels of CAR are associated with higher 30-day mortality. Despite a moderate sensibility, considering the low cost and the predictivity of CAR, it should be considered a standard predictive marker.

## 1. Introduction

Hip fractures are a frequent cause of hospitalization in the elderly population [1] and the incidence is projected to grow from 1.7 million cases in 1990 to 6.3 million cases in the world by 2050 [1,2]. In the United States, there are approximately 150.000 hip fractures per year, representing a significant financial burden with an annual cost between USD 10.3 and USD 15.2 billion [3,4]. In addition, hip fractures are associated with a high incidence of postoperative complications and early mortality within 30 days [5,6,7].

Age, gender, and pre-existing comorbidities have been described as the main non-modifiable risk factors for worse outcomes, including death, after hip fracture [8]. According to Khan et al. [9], patients surgically treated within 48 h from admission have reported a lower 30-day mortality rate compared to patients treated after 48 h at 4% and 11%, respectively (*p* = 0.006). In a recent systematic review and meta-analysis of 28 prospective observational studies with 31.242 patients [10], it was reported that treatment within 48 h from admission was associated with a 20% lower risk of 1-year mortality (RR 0.80, 95%CI 0.66–0.97). In order to predict the 30-day mortality rate after hip fracture surgery, multiple serum markers have been investigated [11,12]. Sheickh et al. [11], reported a 30-day mortality rate of 8.7% (1356 patients) showing that hemoglobin levels below 10 g/dL were significantly associated with increased mortality (*p* < 0.003). In addition, an elevation of lactate level at the time of admission of ≥1 mmol/L was significantly associated with higher 30-day mortality (OR 1.9, 95%CI 1.5–2.3) [12].

Increased levels of C-reactive protein (CRP) and low levels of serum albumin have been associated with poor prognosis and increased mortality after hip fracture [13,14,15,16]. Moreover, the ratio between preoperative CRP and albumin (CAR), a combination of markers for systemic inflammation and nutritional status, has been proposed as a good predictor of mortality in multiple medical settings including patients affected by sepsis, septic shock [17,18], and acute coronary syndrome [19] and critically ill and oncologic patients [20,21]. C-reactive protein (CRP) and albumin are commonly used parameters for the measurement of the activity of inflammatory conditions and are known as positive and negative acute phase reactants (APRs). The CRP/albumin ratio is determined by dividing the CRP by the albumin measurement and is an established scoring system used to determine the degree and activity of inflammatory disease, which is a more useful indicator of the status of inflammation than CRP or albumin alone [22].

To date, only a few studies have examined the predictive models for 30-day mortality after hip fractures [22,23], however, to the best of our knowledge, the use of CAR as a predictive marker has not been thoroughly investigated [24,25]. The aim of this study was to establish if an association exists between high levels of CAR and 30-day mortality after hip fractures. In addition, in order to clarify if CAR can be considered a valuable predictive marker of early mortality, a Receiving Operating Characteristics curve (ROC) was reported. Furthermore, we evaluated the minor and major 30-day complications rate and its relationship with CAR values.

## 2. Material and Methods

The study was performed in accordance with the ethical standards in the 1964 Declaration of Helsinki and with the HIPAA regulation. The Institutional Review Board (IRB) of the author’s institution defined this study as exempt from IRB approval as all patients received standard care as per the treating center and the surgeon’s usual practice. A retrospective observational study was performed on 839 patients. All patients who underwent surgical treatment for hip fracture between January 2006 and December 2018 at two institutions (Orthopaedic Institute of Southern Italy Franco Faggiana, Reggio Calabria, and Franco Scalabrino, Messina, Italy) were initially included. Data regarding baseline characteristics, injury mechanisms, trauma energy content, and fracture type classified according to the AO Foundation/Orthopaedic Trauma Association (AO/OTA) classification system [26], time and date of radiographic examination, and details on treatment were initially collected.

### 2.1. Study Population

Inclusion criteria were all patients with a hip fracture classified according to the AO/OTA classification system and with a minimum follow-up of 30 days. Exclusion criteria were patients younger than 65 years, pathologic fractures secondary to neoplasia, fractures secondary to high-energy trauma, and previous surgery within 6 months before admission. To avoid dependency issues, patients with simultaneous bilateral, multiple fractures (polytrauma), and periprosthetic fractures after hip/knee arthroplasty were excluded. After the application of inclusion and exclusion criteria, 676 patients were included in the final analysis. The mean age was 82.3 ± 10.2 years (range, 65–103 years). Demographic and clinical data were extracted from the institutional database. For each patient, age at the time of surgery, gender, body mass index (BMI), and Charlson Comorbidity Index (CCI) were collected [27].

### 2.2. Serum Markers Measurement

As part of the pre-operative assessment, serum markers, including CRP, Albumin, and creatinine levels were measured. All laboratory data were collected by venous blood sampling the same day or the day before surgery. In the case of multiple blood samples, the one closest to the surgery was used. The CRP to Albumin ratio was calculated as (CRP/Albumin) × 100, while the estimated filtration glomerular rate (eFGR) was calculated based on the modified diet in renal disease (GFR (mL/min/1.73 m^2^) = 175 × (S_cr_)^−1.154^ × (Age)^−0.203^ × (0.742 if female) × (1.212 if African American) (conventional units)) [28].

### 2.3. Surgical Protocols

All surgical interventions were performed or supervised by a consultant orthopedic surgeon. In 434 cases (out of 676, 64.2%), the fracture was a 3.1A1-3 type (trochanteric region fracture); in 166 cases (24.6%), it was a 3.1B2/3 (transcervical and basicervical fracture); and in 76 cases (11.2%), it was a 3.1B1 type (subcapital fracture). In the case of fractures classified as 3.1A1.2 (two-part fracture), 3.1A1.3 (lateral wall intact), 3.1A2.2 (intermediate fragment), and 3.1A2.3 (with 2 or more intermediate fragments), the surgical indication was closed reduction and internal fixation (CRIF) with an intramedullary nail (Basic Nail, Gruppo Bioimpianti, Italy). Surgery was performed on a traction table with the patient in the supine position. In the case of subtrochanteric fractures (3.1A3.1, 3.1A3.2, 3.1A3.3), the surgical indication was a modular tapered titanium stem (Profemur-R, Microport int., Arlington (TN), United States of America) using a posterior approach. In the case of 3.1B and 3.1C fractures, hemiarthroplasty or total hip arthroplasty (THA) was performed through a posterior approach. Surgical treatment was performed within 48 h of hospital admission in 648 (out of 676, 95.4%). If it was not possible to perform surgery in the first 48 h (28 out of 676, 4.6%), it was carried out within 72 h of admission. All patients received prophylactic antibiotics and deep vein thrombosis (DVT) prophylaxis with low-molecular-weight heparins (LMWH) and compression stockings for 4 to 6 weeks after the surgical procedure. A standardized rehabilitation protocol was used for all patients, with active mobilization on postoperative day 1 (POD1).

#### Post-Operative Outcomes

Here, 30-day mortality was calculated as the standard endpoint. In addition, early postoperative complications, considered to be those encountered within 30 days from admission, were collected. For the purpose of this study, urinary tract infection (UTI), pneumonia, acute kidney injury (AKI), delayed wound healing, and superficial infections were considered minor complications. Deep infections, myocardial infarction (MI), cardiac arrest, unplanned intubation, sepsis, pulmonary embolism (PE), and death were considered major complications.

### 2.4. Statistical Analysis

Categorical variables were presented as absolute frequency and percentage. Continuous variables were presented as mean ± standard deviation (SD). The demographic and clinical characteristics of the two groups, survivors and non-survivors, were compared using Pearson’s Chi-Square test and the Student’s *t*-test, respectively, for categorical and continuous variables. A univariate analysis was performed for logistic regression analysis to evaluate the correlation of CRP/Albumin ratio and other clinical characteristics. The diagnostic discrimination of independent predictors of mortality and minor and major complications for CRP/Albumin ratio was assessed with the ROC curves. The Youden index method was used to determine the prediction cut-off value of the CRP/albumin ratio. Statistical significance was indicated by *p* < 0.05.

## 3. Results

### 3.1. Demographic Data of Included Patients

The mean age of the patients was 82.3 ± 10.3 years, BMI was 28.3 kg/m^2^ (+/− 3.2 kg/m^2^), and 55.9% of the patients were female. The most common fracture type was 3.1A (64.2%), followed by 3.1B2/3 (24.6%), and 3.1B1 (11.2%). The mean pre-operative CRP level was 111 ± 76.6 mg/dL, the mean preoperative albumin level was 2.9 ± 0.36 g/dL, and the mean preoperative CAR was 21.4 ± 20.1. The mean preoperative creatinine level was 1.7 ± 0.92 mg/dL, and the mean eGFR was 69.2 ± 29.7 mL/min/1.73 m^2^. The incidence of early postoperative minor complications was 8.4%, early major complications were 5.4%, and the 30-day mortality rate was 7.9%. (Table 1).

### 3.2. Comparison of Preoperative Data and Outcome of Survivors and Non-Survivors

The mortality rate at 30 days from admission was 7.9% (54 of 676 patients), with a mean age of 88.1 ± 6.2 years. Mean age at the time of surgery (*p* < 0.001), early surgical treatment within 48 h from admission (*p* < 0.001), early minor complications rate (*p* = 0.023), preoperative CRP values (*p* < 0.001), preoperative creatinine values (*p* < 0.001), eGFR (*p* = 0.002) and CAR (*p* < 0.001) were significantly associated with 30-day mortality. (Table 2).

### 3.3. Early Minor and Major Complications

Minor complications at 30 days from admission were reported in 7.2% of the cases (49 of 676 patients). UTI was the most frequent in 21 cases (3.1%), followed by superficial wound infections (1.9%, 13 cases), and delayed wound healing (1.2%, 8 cases). All superficial wound complications were successfully treated with oral broad-spectrum antibiotic therapy and did not require additional surgery. Major complications at 30 days from admission were reported in 5.2% of the cases (35 of 676 patients). The most frequent was PE (2.1%, 14 cases), followed by myocardial infarction (1.6%, 11 cases), and cardiac arrest (0.6%, 4 cases) (Table 3).

### 3.4. CRP to Albumin Ratio as Early Mortality Predictor

**Figure 1:**
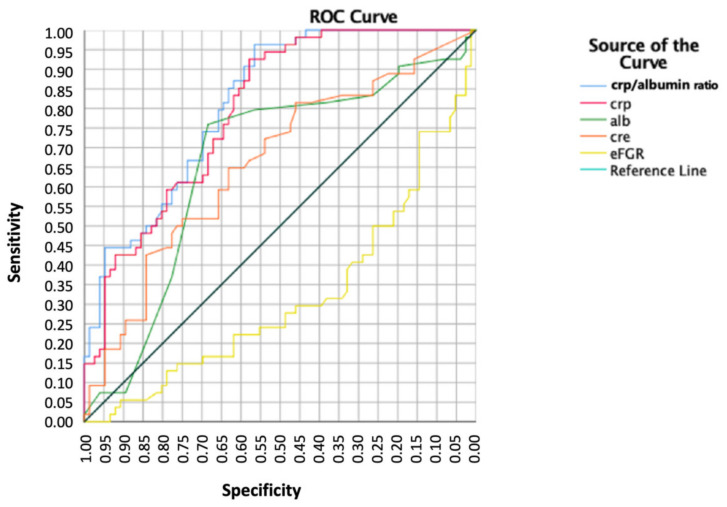
Comparison of ROC curved for CAR, CRP, Albumin, creatinine and eFGR.

**Figure 2:**
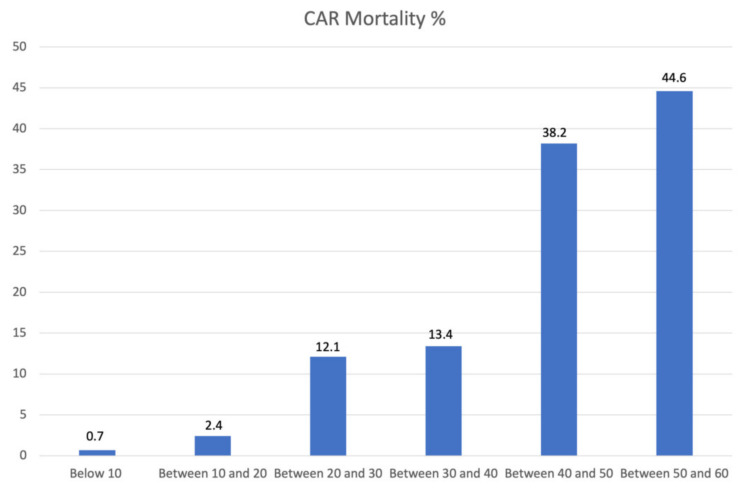
The percentage of mortality for the increasing value of CAR. There is a direct relationship between the incidence of 30-day mortality and the increasing value of CAR. The incidence of early mortality in patients with an average CAR above 10 is significantly lower when compared with patients with a higher CAR (*p* < 0.001).

**Figure 3:**
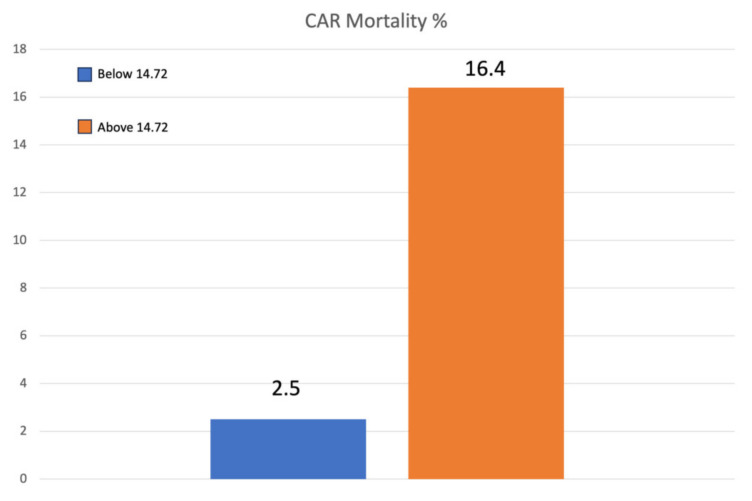
Early mortality (within 30 days of admission), stratified for patients with an average CAR above and below 17.42. Patients with a car below the cut-off value reported a mortality rate of 2.6%, while above the cutoff value was 16.4%.

The mean CAR value was significantly higher in the case of 30-day mortality (37.4 ± 25.6 vs. 14.2 ± 13.8; *p* < 0.001). The AUROC was 0.816 (95% CI, 0.746–0.886). The point of the ROC curve corresponding to the value of 14.72, was associated with a specificity of 87% and sensibility of 40.8% and considered the cut-off (Figure 1). When analyzing values higher than the cut-off, the 30-day mortality rate was 17.9% compared to 1.8% for lower values (Figure 2). The odds ratio for 30-day mortality for patients above and below the cut-off of 14.72 was 9.38 (95% CI; 4.5 to 19.5) (Table 4 and Table 5, Figure 3). For minor complications rate, the AUROC was 0.416 (95% CI; 0.325–0.543), and for major complications, it was 0.484 (95% CI; 0.361–0.557). The CAR value was found to be significantly associated with age (r = 0.247, *p* = 0.004) and CRP (r = 0.86, *p* < 0.001), while no such relationship was reported for eGFR (r = 0.087, *p* = 0.327), serum albumin levels (r = 0.53, *p* = 0.55), and creatinine levels (r = 0.025, *p* = 0.779).

## 4. Discussion

The CAR level at the time of admission in patients affected by hip fractures was significantly related to 30-day mortality. CAR was a reliable predictor of mortality based on the ROC curve with an AUROC value of 0.816 with a cut-off value of 14.72 being predictive of early mortality with a high specificity (87%) and a moderate sensibility (40.8%). Considering our results, CAR should be routinely used as a predictive factor of 30-day mortality in patients > 65 years of age affected by hip fractures. Our results are in line with Capkin et al. [24], reporting that a CAR > 2.49 is a strong predictor for 1-year mortality in patients who undergo hemiarthroplasty due to hip fracture.

High preoperative levels of serum CRP (>10 mg/dL) [13,29] and low levels of serum albumin (<3 g/dL) [14,30] are widely considered independent predictors of mortality after hip fracture in the elderly population. Currently, preoperative CRP is used to predict early and late mortality after femoral fractures, but the results are still inconsistent [13,29]. Marino et al. [13], reported that all patients affected by hip fractures have increased levels of CRP (>3 mg/dL). According to Chapman et al. [29], CRP levels > 500/d (d = number of postoperative days) are related to an increased risk of complications and mortality (10% vs. 3.9%; RR = 2.74). In addition, Fakler et al. [30] and Kim et al. [18] reported that preoperative CRP levels > 10 mg/dL are correlated with increased 1-year mortality and that patients with CRP levels > 40 mg/dL have a higher risk of mortality compared with patients with low to mild inflammatory response (up to 40% in patients with high inflammatory response) [30]. Beloosesky et al. [31], documented that patients with levels of CRP > 15 mg/dL for more than 48–60 h after surgery have a higher risk of complications, but not higher 6-month mortality. In addition, CRP levels > 10 mg/dL are related to a 30-day mortality rate [32]. Conversely, Niessen et al. [14], reported that preoperative CRP levels were not associated with an increased 12-month mortality rate.

According to the current literature, serum concentrations of albumin have been related to 1-year mortality after hip fracture [15,16,33]. Hypoalbuminemia is defined as a serum concentration of albumin < 3.5 g/dL and is a common condition in more than 50% of elderly patients affected by a hip fracture [15,34]. Bohl et al. [15], reported significantly higher 1-year mortality in patients with hypoalbuminemia compared to patients with normal albumin serum concentration, 9.9% and 5.5%, respectively (*p* < 0.005). Similarly, a series of retrospective studies reported that preoperative hypoalbuminemia was associated with increased 1-year mortality after hip fracture [16,33].

According to our experience, CAR would be a valuable prognostic factor when considering 30-day mortality after a traumatic hip fracture considering that it provides information on the inflammatory and nutritional status of the patients [17,23]. Ranzani et al. [17], reported that CAR is a better predictive marker for 90-day mortality than CRP alone in patients with severe sepsis or septic shock. Similarly, Kim et al. [18], reported that CAR was a reliable predictor for 180-day mortality in septic shock patients treated with Early Goal-Directed Therapy (CAR cut-off value of 5.9). Duman et al. [19], reported that CAR can be used as a reliable predictor for thrombus burden in patients with acute coronary syndrome. In addition, high levels of CAR have been associated with increased in-hospital mortality, and with an increased 30-day mortality rate in general surgery, and critically ill patients [20,21]. Moreover, high CAR levels have been successfully used as a reliable predictor of mortality in different oncologic settings [22,23]. In patients affected by pancreatic cancer, CAR is currently used as an independent factor of poor long-term prognosis after surgical resection [22,35], and a valuable predictor for advancement in non-operable pancreatic cancer [36]. Similarly, CAR is a more reliable predictor of mortality than other serum markers in patients affected by lung cancer [37], in small lung cell cancer, non-small lung cell cancer, and adenocarcinoma [23,38,39,40].

There were multiple limitations to this study. It was a retrospective observational study with all the limitations being related to the design of the study. In addition, stratification of the patients based on the etiology of the fracture, the type of fracture, or the type of treatment was not performed, leading to difficulty in the interpretation of the results and introducing a possible bias in the generalization of the results. Moreover, the lack of randomization and blinding of outcome assessments may have led to a high risk of selection and detection bias, respectively. However, to our knowledge, this was the first study that used CAR as a predictive marker for 30-day mortality after surgical treatment of a hip fracture.

In conclusion, preoperative levels of CAR > 14.72 are predictive of increased 30-day mortality rates in patients > 65 years of age affected by hip fractures with a high specificity of 87% and a relatively moderate sensitivity of 40.7%. In the authors’ opinion, considering the low costs and good predictive results of CAR for 30-day mortality, large multicenter and national database studies would be necessary in order to better define, and eventually confirm, the preliminary results of this marker.

## Figures and Tables

**Table 1:** Baseline demographic features of patients that were surgically treated for hip fracture between 2006 and 2018.

Variables	Total	Trochanteric Region 31A	Femoral Neck Fracture 31B2-3	Subcapitis Fracture 31b1
N° of patients	676	434 (64.2%)	242 (35.8%)	76 (11.2%)
Gender (female %)	378 (55.9%)	251 (58.4%)	127 (51.8%)	41 (54%)
Age (+/− SD)	82.3 +/− 10.3	81.3 +/− 8.4	83.3 +/− 10.7	84.3 +/− 11.5
BMI (Kg/m^2^)	28.3 +/− 4.2	27.9 +/− 3.8	29.1 +/− 4.3	28.1
ASA Score	3.6 +/− 1.1	3.7 +/− 1.3	3.1 +/− 1.2	3.9
Surgery within 48 h (%)	648 (95.4%)	422 (97.3%)	226 (93.4%)	71 (93.4%)
Time of surgery (min)	55 +/− 19	32 +/− 14	53 +/− 20	47 +/− 22
Charlson Comorbidity Index	6.11 +/− 1.8	6.07 +/− 1.9	5.99 +/− 1.6	6.44 +/− 1.7
Minor complications	57 (8.4%)	36 (8.9%)	18 (7.2%)	6 (7.9%%)
Major complications	35 (5.2%)	23 (5.3%)	12 (4.8%)	4 (5.3%)
Death	54 (7.9%)	33 (7.6%)	21 (7.8%)	8 (10.5%)
CRP (mg/dL)	111 +/− 76.6	107.5 +/− 72.2	112.8 +/− 75.6	113.4 +/− 78.1
Albumin (g/dL)	2.9 +/− 0.36	2.8 +/− 0.32	2.83 +/− 0.38	3.3 +/− 0.41
CRP/Albumin Ratio	21.4 +/− 20.1	20.3 +/− 19.4	25.1 +/− 18.3	23.3 +/− 17.4
Creatinine (mg/dL)	1.7 +/− 0.92	1.6 +/− 0.84	1.7 +/− 0.91	1.8 +/− 0.94
eGFR	69.2 +/− 29.7	67.5 +/− 27.4	70.1 +/− 28.9	70.2 +/− 30.7

BMI = body mass index; ASA = American Society of Anesthesiology, CRP = C-reactive protein, eGFR = estimated glomerular filtration rate.

**Table 2:** Demographic and clinical information: survivors versus non-survivors.

Variables	Total (Survivors + Non Survivors	Survivors	Non-Survivors	*p*-Value	Significant
N° of patients	676	622 (92.1%)	54 (7.9%)	//	//
Gender (female %)	378 (55.9%)	350 (56.3%)	28 (51.9%)	0.53	Not significant
Age (+/− SD, years)	82.3 +/− 10.3	78.7 +/− 10.6	88.1 +/− 6.1	<0.001	Significant
BMI (Kg/m^2^)	28.3	28.1	28.5	0.48	Not Significant
ASA Score	3.6	3.3	3.8	0.13	Not Significant
Surgery within 48 h (%)	648 (95.8%)	606 (97.4%)	42 (77%)	<0.001	Significant
Time of surgery (min)	55 +/− 19	53.4 +/− 18	55.8 +/− 19	0.58	Not Significant
Charlson Comorbidity Index	6.11 +/− 1.8	5.98 +/− 1.5	6.18 +/− 1.9	0.22	Not Significant
Minor complications	57 (8.4%)	49 (7.2%)	8 (14.8%)	0.023	Significant
Major complications	35 (5.2%)	31 (4.9%)	4 (7.4%)	0.13	Not Significant
Death	54 (7.9%)	0 (0%)	54 (100%)	<0.001	significant
CRP (mg/dL)	111 +/− 76.6	41.2 +/− 38	111 +/− 76.1	<0.001	Significant
Albumin (g/dL)	2.9 +/− 0.36	2.8 +/− 0.34	2.9 +/− 0.37	0.34	Not Significant
CRP/Albumin Ratio	21.4 +/− 20.1	14.2 +/− 13.8	37.4 +/− 25.6	0.0002	Significant
Creatinine (mg/dL)	1.7 +/− 0.92	0.98 +/− 0.57	1.29 +/− 0.82	0.0003	Significant
eGFR (mL/min/1.73 m^2^)	69.2 +/− 29.7	79.1 +/− 27.3	44.8 +/− 20.1	0.002	Significant

BMI = body mass index; ASA = American Society of Anesthesiology, CRP = C-reactive protein, eGFR = estimated glomerular filtration rate.

**Table 3:** Minor, major complications, and mortality within 30 days of hip fractures.

Complications (n, %)	31A (n, %) 434	31B2-3 (n, %) 166	31B1 (n, %) 76
Minor Complications (54, 7.9%)	38, 8.7%	10, 6.2%	6, 7.8%
Urinary tract infection (21, 3.1%)	13, 2.9%	6, 3.6%	2, 2.6%
Superficial Infection (13, 1.9%)	7, 1.6%	3, 1.8%	3, 3.9%
Delayed wound healing (8, 1.2%)	4, 0.9%	2, 1.2%	2, 2.6%
Acute kidney Injury (7, 1%)	3, 0.7%	2, 1.2%	2, 2.6%
Major Complications (35, 5.2%)	21, 4.8%	9, 5.4%	5, 6.5%
Pulmonary embolism (14, 2.1%)	8, 1.8%	3, 1.6%	3, 3.9%
Myocardial infarction (11, 1.6%)	6, 1.4%	3, 1.8%	2, 2.6%
Cardiac arrest (4, 0.6%)	2, 0.5%	1, 0.6%	1, 1.3%
Sepsis (3, 0.4%)	2, 0.5%	1, 0.6%	-
Unplanned Intubations (3, 0.4%)	2, 0.5%	1, 0.6%	-

**Table 4:** Values of AUC, std. error and 95% CI of CAR, CRP, albumin, creatinine, and eGFR. CAR was demonstrated to be the better predictor for 30-day mortality when compared with the other factors.

Variables	Area under the Curve	Std. Error	95% CI
CRP/Albumin Ratio	0.816	0.036	0.746/0.886
CRP	0.797	0.038	0.723/0.871
Albumin	0.663	0.05	0.565/0.761
Creatinine	0.660	0.049	0.565/0.756
eGFR	0.319	0.048	0.225/0.413

CRP = C-reactive protein, eGFR = estimated glomerular filtration rate.

**Table 5:** Odds ratio for 30-day mortality associated with a CAR value above and below the cutoff value of 14.72.

Statistical Measure	CRP to Albumin Ratio > 14.72	
	30 Days Mortality	95% CI
Odds Ratio	9.38	14.5 to 19.5
Sensitivity	83.33%	70.71% to 92.08%
Specificity	69.45%	65.67% to 73.05%
Positive Likelihood Ratio	2.73	2.31 to 3.23
Negative Likelihood Ratio	0.24	0.13 to 0.44
Disease prevalence	7.99%	6.06% to 10.29%
Positive Predictive Value	19.15%	16.68% to 21.89%
Negative Predictive Value	97.96%	96.35% to 98.87%
Accuracy	70.56%	66.97% to 73.98%

## Data Availability

Non-digital data supporting this study are curated at Orthopaedic Institute of Southern Italy “Franco Scalabrino”, Messina, 98165, Italy.
